# Gold Nanoparticle Mediated Laser Transfection for Efficient siRNA Mediated Gene Knock Down

**DOI:** 10.1371/journal.pone.0058604

**Published:** 2013-03-11

**Authors:** Dag Heinemann, Markus Schomaker, Stefan Kalies, Maximilian Schieck, Regina Carlson, Hugo Murua Escobar, Tammo Ripken, Heiko Meyer, Alexander Heisterkamp

**Affiliations:** 1 Biomedical Optics Department, Laser Zentrum Hannover e.V., Hannover, Germany; 2 Department of Pediatric Pneumology, Allergy and Neonatology, Hannover Medical School, Hannover, Germany; 3 Small Animal Clinic, University for Veterinary Medicine, Hannover, Germany; 4 Department of Cardiothoracic, Transplantation and Vascular Surgery, Hannover Medical School, Hannover, Germany; 5 Institute of Applied Optics, Friedrich-Schiller-University Jena, Jena, Germany; University of Toronto, Canada

## Abstract

Laser based transfection methods have proven to be an efficient and gentle alternative to established molecule delivery methods like lipofection or electroporation. Among the laser based methods, gold nanoparticle mediated laser transfection bears the major advantage of high throughput and easy usability. This approach uses plasmon resonances on gold nanoparticles unspecifically attached to the cell membrane to evoke transient and spatially defined cell membrane permeabilization. In this study, we explore the parameter regime for gold nanoparticle mediated laser transfection for the delivery of molecules into cell lines and prove its suitability for siRNA mediated gene knock down. The developed setup allows easy usage and safe laser operation in a normal lab environment. We applied a 532 nm Nd:YAG microchip laser emitting 850 ps pulses at a repetition rate of 20.25 kHz. Scanning velocities of the laser spot over the sample of up to 200 mm/s were tested without a decline in perforation efficiency. This velocity leads to a process speed of ∼8 s per well of a 96 well plate. The optimal particle density was determined to be ∼6 particles per cell using environmental scanning electron microscopy. Applying the optimized parameters transfection efficiencies of 88% were achieved in canine pleomorphic adenoma ZMTH3 cells using a fluorescent labeled siRNA while maintaining a high cell viability of >90%. Gene knock down of d2-EGFP was demonstrated and validated by fluorescence repression and western blot analysis. On basis of our findings and established mathematical models we suppose a mixed transfection mechanism consisting of thermal and multiphoton near field effects. Our findings emphasize that gold nanoparticle mediated laser transfection provides an excellent tool for molecular delivery for both, high throughput purposes and the transfection of sensitive cells types.

## Introduction

Laser transfection of cells is a growing research field in the area of molecule delivery as an alternative to established transfection methods like lipofection or electroporation. Several studies have proven the suitability of femtosecond laser pulses for cell membrane perforation [1]–[6]. In most cases the manipulation laser is focused through the objective of an inverse microscope onto the cell surface to create a spatially confined pore, which allows the diffusion of extracellular molecules into the cytoplasm. Using this approach, delivery of different molecules into cell lines, primary cells and stem cells has been demonstrated [1]–[3]. It allows efficient and gentle transfection of cell lines as well as sensitive cell types, but bears some major disadvantages: The used femtosecond laser systems are expensive, bulky and their operation needs a high degree of technical expertise. In addition the applied mode of action only allows manipulation of single cells as the laser focus has to be carefully aligned to the membrane of each cell. Lately, several approaches have tried to overcome this drawback by the use of non-diffractive laser beams [7], micro fluidic channels [8] or implementation of a spatial light modulator to create multiple foci [9]. So far, none of these approaches can provide an efficient, fast and intuitively usable transfection platform. As a consequence, although being quite successful for single cell applications, laser based transfection has not reached broad routine usage to date.

In order to combine the mentioned advantages of laser transfection with a high throughput, we describe a technique termed gold nanoparticle mediated (GNOME) laser transfection: The cells are incubated with gold nanoparticles (AuNP) with a diameter of 200 nm. Due to sedimentation the AuNP attach to the cell membrane. The sample is then irradiated by a weakly focussed laser beam. The particle-laser interaction leads to plasmon resonances on the particles. These induce thermal and near field effects, which in turn can evoke transient cell membrane permeabilization, enabling diffusion of extracellular molecules into the cytoplasm. This allows a drastically enhanced throughput compared to optoinjection due to possible high speed scanning of the sample, increased spot diameter and the automated setup. Another benefit is the improved usability and reduced cost expenditure of the experimental setup. Due to its physical approach GNOME laser transfection allows cell type independent delivery of a large variety of molecules.

Schomaker et al. demonstrated proof of principle of this approach for the delivery of small fluorescent dyes, fluorescent labeled siRNA and plasmid DNA [10], [11]. Another recent study demonstrated the feasibility for the transfection of human melanoma cancer cells, reaching a transfection rate of 23% [12]. The authors applied an off-resonant (800 nm) femtosecond laser system and suppose plasma induced nanocavitation to be the perforation mechanism [12], [13]. In contrast, the pioneering studies of Yao et al. used small (15 & 30 nm), strong absorbing antibody-gold conjugates. They demonstrated delivery of 10 kDa fluorescein isothiocyanate (FITC)-dextran derivatives and an AlexaFluor488 labeled MIB-1 antibody [14], [15]. Furthermore, Lukianova-Hleb et al. were able to transfect cells by the use of plasmonic nanobubbles created around AuNP clusters within the target cells [16].

Other approaches applied absorbing nano- and microparticles consisting of other materials than gold. By irradiating cells incubated with latex microparticles Umebayashi et al. showed delivery of fluorescein diacetate into cells [17]. Chakravarty et al. demonstrated that irradiation of carbon black nanoparticles induces the so called carbon-steam reaction, which evokes membrane permeabilization by acoustic shock waves [18].

For the gold nanoparticle mediated approach, the membrane permeabilization might be explained by two basic mechanisms: Near field enhancement and particle heating [19]. The amount in which these contribute to the biological effect is highly dependent on the experimental parameters, mainly AuNP diameter, wavelength and pulse duration.

The near field enhancement arises because the incident electromagnetic field induces collective oscillations of the quasi-free electrons of the AuNP, the so called surface plasmons. The formed multipoles of oscillation [20] induce an electromagnetic field in close vicinity to the particle surface, which can be much higher than the incident field [21], [22]. Under these conditions the creation of a low-density plasma and subsequent ionisation of molecules or even optical breakdown are likely to occur [12], [23], [24].

Thermal heating is caused by the absorption of incident light: On a timescale of less than 100 fs the excitation by a laser pulse leads to a non-thermal, energetic distribution of the conduction electrons within the AuNP [25], [26]. Within the following picoseconds the non-thermal distribution decays to a thermal distribution by electron-electron scattering and the thermal energy is submitted onto the lattice by electron-phonon scattering [27]. Depending on the extent of heating, a number of subsequent effects can occur, ranging from the denaturation of proteins in the closer proximity to the AuNP, to fragmentation and evaporation of the AuNP [28], [29].

**Figure 1:**
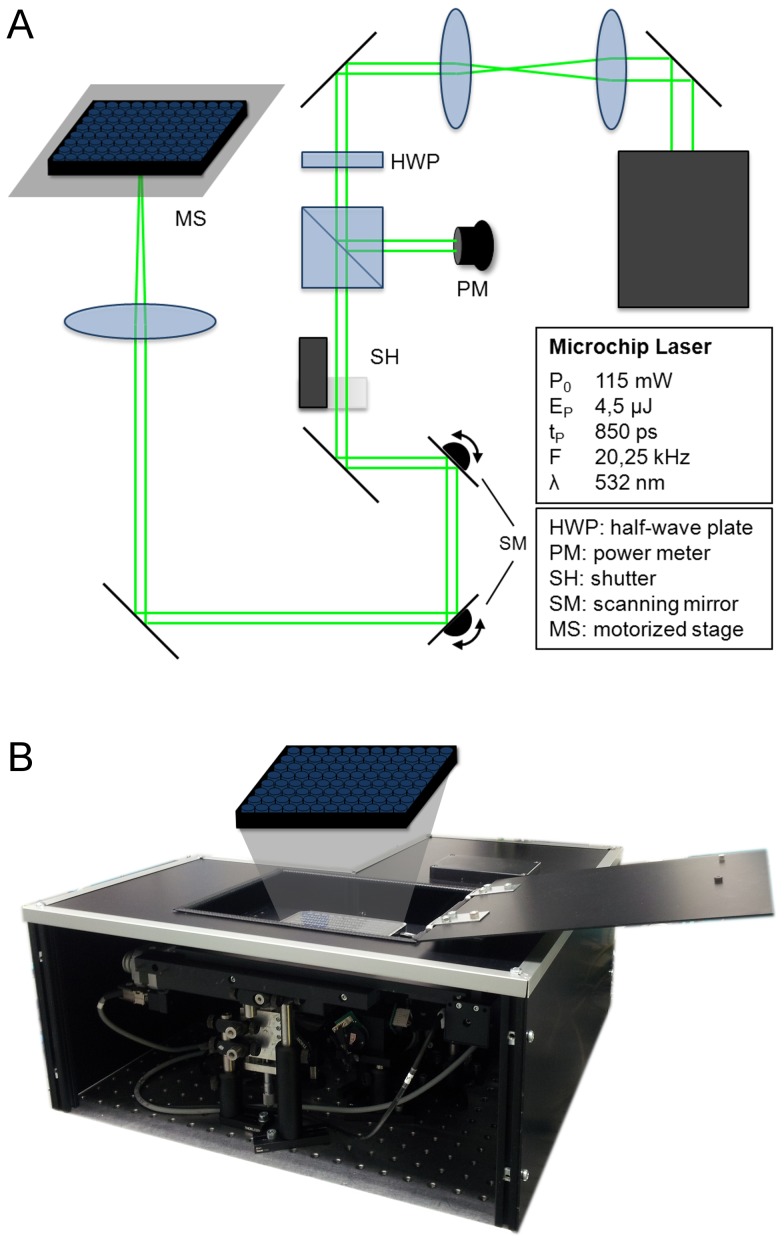
Experimental setup. A: Schematic drawing and B: photograph of the setup.

Herein, we utilized an automated experimental setup, which allows fast and convenient selection of different parameters, offers complete laser safe operation and is therefore usable outside of specialized laser labs (Fig. 1). Our study provides detailed information about the optimal parameter regime for GNOME laser transfection, taking into account radiant exposure, scanning velocity and AuNP concentration. Environmental scanning electron microscopy (ESEM) was applied to determine the actual amount of AuNP that are attached to the cells. The cytotoxicity of the method has been evaluated by a Resazurin based assay and followed up over 96 h. The efficiency of siRNA transfection was determined by employing fluorescent labeled siRNA and FACS analysis. We demonstrated for the first time a functional gene knock down by means of GNOME laser transfection. The knock down was validated via Enhanced Green Fluorescent Protein (EGFP)-fluorescence depletion after siRNA transfection and western blot analysis. Furthermore, we give indications for the nature of the perforation mechanism.

## Materials and Methods

### Experimental Setup

The optical transfection setup utilized a 532 nm Nd:YAG microchip laser (Horus Laser, Limoges, France) emitting 850 ps pulses at a repetition rate of 20.25 kHz. The beam diameter was adjusted using a telescope. The laser power was reduced by a combination of a half-wave plate and a polarizing beam-splitter (Thorlabs, Newton, USA). A galvanometer scanner (Müller Elektronik, Spaichingen, Germany) allowed scanning of the laser spot over the sample. A motorized stage (Carl Zeiss, Jena, Germany) allowed positioning of the sample and automated sequential selection of single wells within a multiwell plate by a self-developed, LabView based software.

### Cell preparation and GNOME laser transfection

Canine pleomorphic adenoma ZMTH3 cells [30] were cultured routinely in RPMI 1640 supplemented with 10% FCS and 1% Penicillin/Streptomycin (all Biochrom AG, Berlin, Germany).

**Figure 2:**
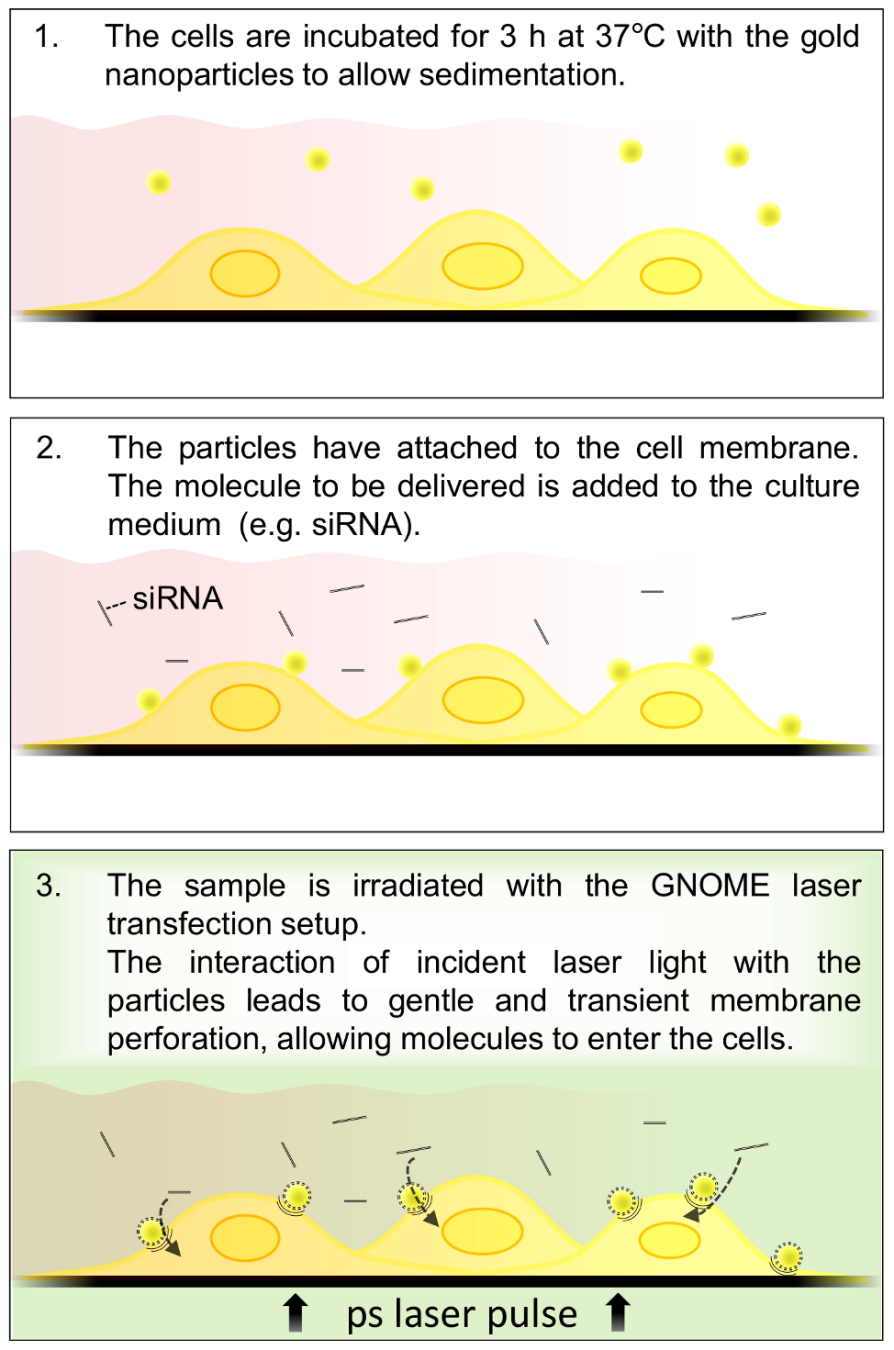
Experimental procedure for GNOME laser transfection. Cells are incubated with AuNP (1), the molecule to be delivered is added (2) and the sample is irradiated to permeabelize the cell membrane (3). Drawings are not true to scale.

For parameter evaluation 2,5×10^4^ ZMTH3 cells per well of a black wall/clear bottom 96 well plate (BD Bioscience, Heidelberg, Germany) were seeded 24 h before transfection. The cells were incubated with 200 nm AuNP (Kisker Biotech, Steinfurt, Germany) solved in culture medium for 3 h at 37°C, then the molecule to be delivered (2 mg/ml 10 kDa FITC-dextran; Sigma-Aldrich, Steinheim, Germany) was added in fresh culture medium and the samples were laser-treated (Fig. 2). Afterwards cells were incubated for 30 min at 37°C and washed three times. In order to determine the viability, 10% (v/v) of the QBlue viability assay kit (BioChain, Newark, USA), a resazurin based, fluorometric metabolism assay, were added to the culture medium and incubated for one hour. Delivery was monitored in a plate reader (Infinite 200 pro, Tecan, Männedorf, Switzerland) at EX488/EM520 nm, the viability was measured at EX570/EM600 nm. The normalized efficiency was calculated by subtracting the fluorescent background from each well and normalizing the values corresponding to the highest value of the dataset.

To evaluate the transfection efficiencies the cells were prepared and incubated with particles as described above. Then 100 µM siRNA labeled with AlexaFluor488 (Qiagen, Hilden, Germany) was added to the samples and the cells were laser-treated. Dead cells were stained with ToPro3 (Invitrogen, Carlsbad, USA). The transfection efficiency was measured by flow cytometry (FACS Calibur, BD Bioscience, Heidelberg, Germany).

### siRNA mediated knock down

For EGFP knock down ZMTH3 cells stably transfected with pd2-EGFP-N1 (Clontech Laboratories, Mountain View, USA) were laser-transfected with anti-GFP siRNA (Qiagen, Hilden, Germany). After 24 h EGFP fluorescence was measured at EX475/EM511 nm and viability assessed as described above. To account for possible cell losses, knock down efficiencies were calculated as
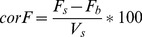
(1)

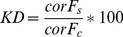
(2)


with corF = corrected fluorescence, F_s_ = fluorescence of the sample, F_b_ = fluorescence of the blank (empty well), V_s_ = viability of the sample, KD = knock down in % and F_c_ = fluorescence of untreated control cells.

### Western blot analysis

48 h after GNOME laser transfection with anti-GFP siRNA all samples and controls were lysed in 40 µl lysis buffer (150 mM NaCl, 1% Triton X-100, 50 mM Tris, pH 8.0, Roche cOmplete ultra protease inhibitor) per well. Protein contents were determined using the Roti Quant universal Kit (Carl Roth, Karlsruhe, Germany). After electrophoresis and blotting GFP was detected by a 1∶4,000 dilution of the Living Colors A.v. Monoclonal Antibody (Clontech Laboratories) and a 1∶1,000 dilution of an anti-mouse-HRP conjugate (dianova, Hamburg, Germany). β-Actin was detected by a 1∶20,000 dilution by a directly HRP linked antibody (dianova).

### Toxicity testings

To assess the viability after laser treatment for longer time scales 5×10^3^ cells per well were seeded and then laser treated as described above. To measure the impact of AuNP incubation, cells were incubated with 0.5 or 5 µg/cm^2^ for 3 or 24 h, respectively. Viability was measured using the QBlue viability assay kit. Afterwards the staining solution was replaced by culture medium and cells were further incubated until the next time point. Cells treated with 12 µg/ml Digitonin (Sigma-Aldrich) served as negative control.

### ESEM Imaging

For ESEM imaging, the cells were grown on cover slides (diameter 12 mm) in a 24 well plate and treated with the indicated concentrations of AuNP for 3 h. The culture medium was removed and the samples were fixed in 4% paraformaldehyde and 2.5% glutaraldehyde (Sigma-Aldrich) for 10 min at room temperature. The samples were carefully rinsed with distilled water and imaged in an electron microscope (Quanta 400 F, FEI, Eindhoven, Netherlands) in wet mode. Images were taken after two purge cycles between 6 and 13 mbar at 2°C, 6 mbar and a high voltage of 15 kV. The cell surface area and particle count were analyzed using ImageJ [31].

### UV/VIS spectra

200 nm AuNP were solved in a concentration of 10 µg/ml in RPMI without phenol red supplied with 10% FCS. For each sample 200 µl AuNP suspension were irradiated in a 96 well. The absorbance was acquired in a UV/VIS spectrometer (UV 1650-PC, Shimadzu, Duisburg, Germany) against RPMI without AuNP.

## Results and Discussion

### Evaluation of transfection parameters

**Figure 3:**
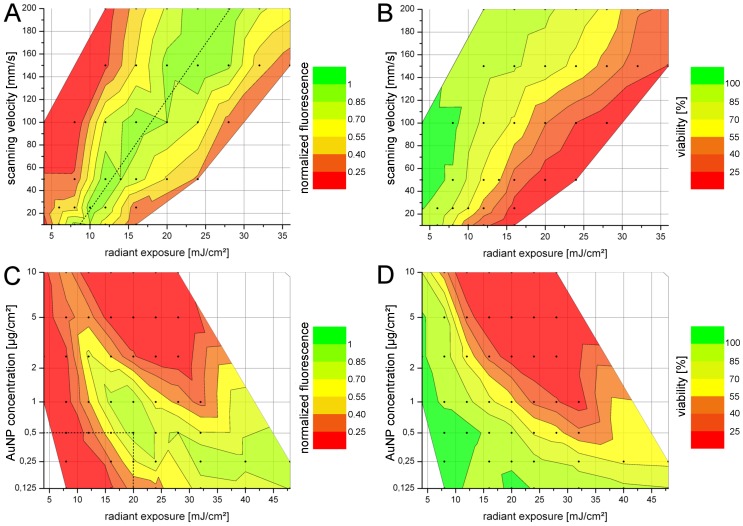
Fluorescence level and viability for different transfection parameters for the delivery of 10 kDa FITC-dextran. A: Fluorescence for different scanning velocities at a constant AuNP concentration of 5 µg/cm^2^. The scanning velocity was set to values between 10 and 200 mm/s, corresponding to 175 and 9 pulses per point, respectively. For all velocities the same fluorescence level could be achieved by using increasing radiant exposure for higher velocities as indicated by the dotted line. B: The corresponding viability dropped with higher radiant exposure and slower scanning velocities. C, D: High AuNP concentrations led to pronounced cell death even at low values of radiant exposure and thus only provide low levels of fluorescence. Most efficient delivery was achieved at 0.5 µg/cm^2^ and 20 mJ/cm^2^ as indicated by the dotted line in C. Scanning velocity was kept constant at 50 mm/s. Data points represent the mean of n = 4 experiments.

In order to determine the optimal perforation parameters, cells were perforated with FITC labeled dextrans with a molecular weight of 10 kDa. This is comparable to siRNA molecules (∼14 kDa), although differences in charge and 3D structure of the molecules must be considered. The fluorescence of a well after treatment was chosen as a marker for successful delivery. Since fluorescence is directly linked to the total amount of molecules delivered to the cell population, it is dependent not only on the efficiency (amount of cells perforated), but also on the number of molecules delivered per cell. Figure 3 represents the normalized fluorescence and the viability for different scanning velocities (Fig. 3a, b) and for different AuNP concentrations (Fig. 3c, d). Both parameters were evaluated against different values of radiant exposure.

For a given scanning velocity the viability dropped with increasing radiant exposure (Fig. 3a). In parallel the fluorescence increased until it reached a maximum, an optimal balance of delivery and cell viability was established. When the radiant exposure was increased above this critical point, the fluorescence dropped because the cell loss surpassed the gain of fluorescence. For increasing scanning velocities the corresponding radiant exposure yielding the maximum fluorescence also increased. All tested scanning velocities revealed a comparable level of fluorescence, indicating that the same efficiency was obtained (dotted line in Fig. 3a). These results imply that the membrane perforation is provoked by cumulative effects of single pulses. As the repetition rate of the laser system (20.25 kHz) yields a temporal distance of 50 µs between two pulses and thermal processes are finished on a ns-timescale [28], [32] the AuNP can be considered as cooled down to the initial temperature until the next pulse. Therefore no thermal accumulation is likely to occur.

The results for different AuNP concentrations and a fixed scanning velocity of 50 mm/s revealed a strong influence of the concentration on the achievable levels of fluorescence (Fig. 3c). Lower concentrations allowed a higher radiant exposure before the loss in viability got dominant. For concentrations above 1 µg/cm^2^ the maximum fluorescence dramatically dropped. At lower concentrations the maximum peak broadened. Within the parameter set tested, the highest fluorescence was obtained at a concentration of 0.5 µg/cm^2^ and a radiant exposure of 20 mJ/cm^2^ (dotted line in Fig. 3c). The corresponding viability was 90%.

**Figure 4:**
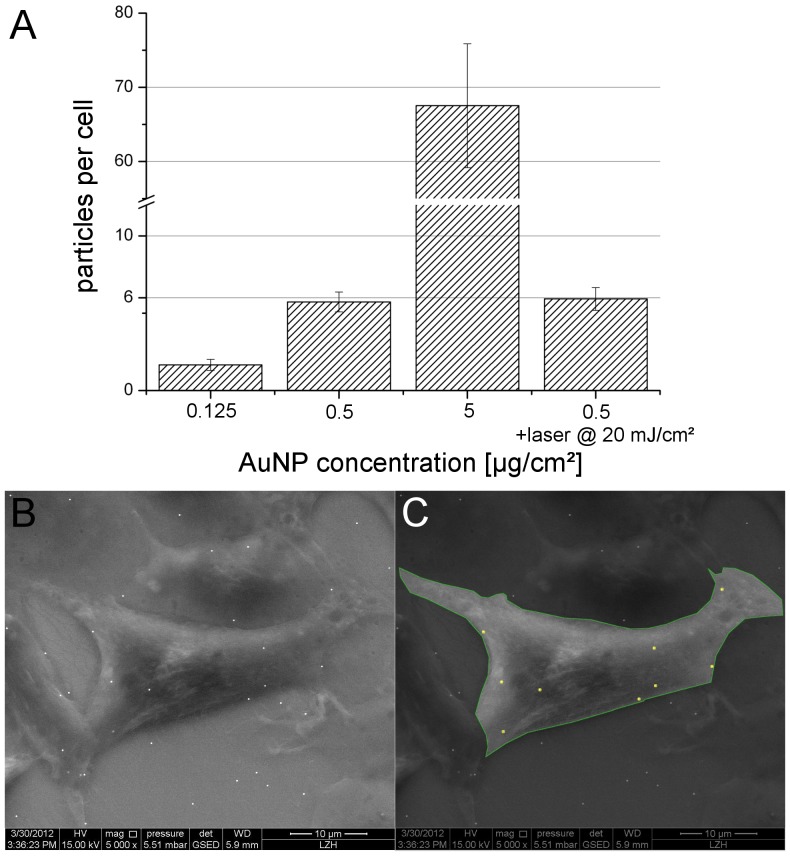
Number of particles per cell. A: Particle count per cell after 3 hours of incubation with different AuNP concentrations. No significant difference in the particle count was observed after irradiation with 20 mJ/cm^2^ (p = 0.46). Values represent the mean of n = 3 experiments ±SEM. B: ESEM image of one exemplary ZMTH3 cell incubated for 3 h with 0.5 µg/cm^2^ 200 nm AuNP in RMPI. C An individual cell was marked and the particles were counted.

To correlate the applied AuNP concentrations with an actual number of AuNP which had attached to a cell, we applied (ESEM) imaging for three different concentrations (Fig. 4b–e). The optimal concentration of 0.5 µg/cm^2^ corresponded to about 6 particles per cell. After irradiation with 20 mJ/cm^2^ no significant difference (p = 0.46) in the particle count was observed. With the lowest concentration tested (0.125 µg/cm^2^), only one particle attached on average per cell.

These findings indicate that few or even single particles per cell are enough for efficient delivery. Larger amounts of particles strongly impair the viability, most likely because the integrity of the cellular membrane is affected to an extent that it cannot reseal after laser treatment. Thus, higher AuNP concentrations have an adverse effect on the efficiency. In contrast, concentrations below 0.25 µg/cm^2^ only led to an incomplete coverage of the cells with particles due to the statistical nature of the attachment process. For 0.125 µg/cm^2^ about 20% of the cells did not bear any particle at all.

Interestingly, AuNP mediated transfection using near infrared femtosecond laser pulses seems to require ∼10-fold or even higher particle concentrations to achieve optimal transfection [10], [12]. Eventually, transfection under these conditions relies on the formation of particle clusters, indicating a different perforation mechanism for the parameters used in our study, which will be discussed below.

### Toxicity of GNOME laser transfection

**Figure 5:**
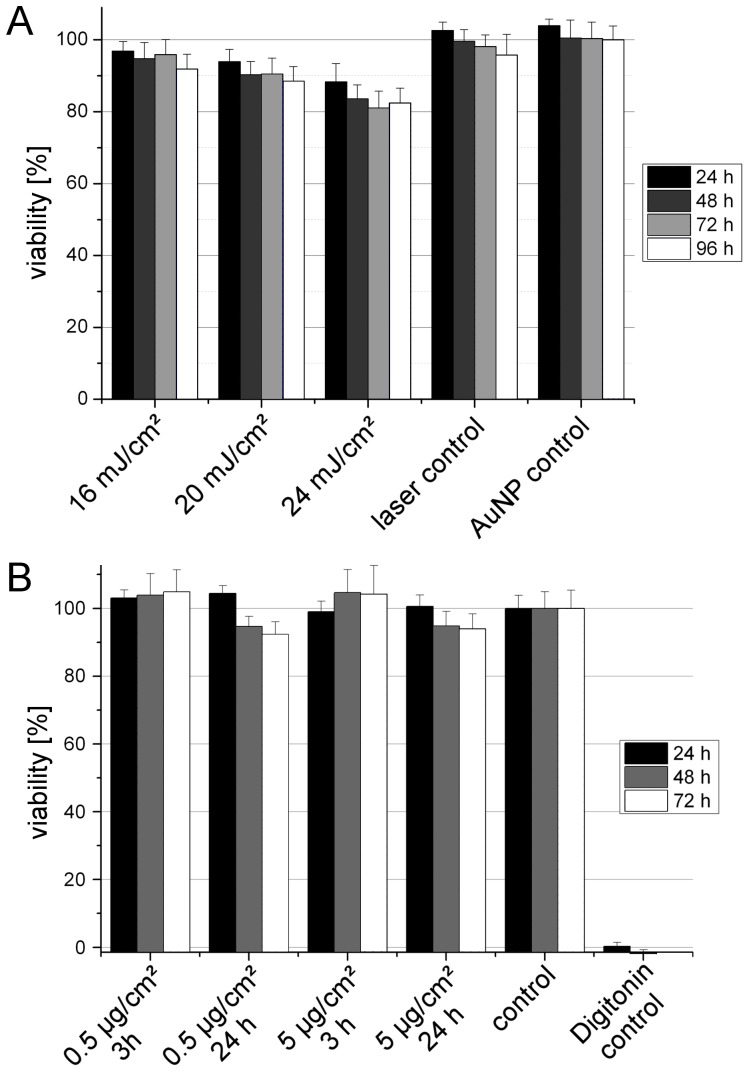
Course of the viability after GNOME laser transfection and AuNP incubation. A: The cells have been incubated with 0.5 µg/cm^2^ 200 nm AuNP and were irradiated with different values of radiant exposure. At the indicated time points after laser treatment the cell viability was measured. For all conditions tested the viability stayed above 80%. B: ZMTH3 cells were incubated with 200 nm AuNP for 3 and 24 h respectively. No significant impact on the viability could be observed after 3 h of incubation. Values represent the mean of n≥3 experiments +SEM.

To evaluate the impact of the established transfection parameters and of the applied AuNP concentrations on the viability at longer time scales, cells were grown for up to 96 h after laser treatment. Figure 5a shows the course of the viability for different values of radiant exposure. Within all tested conditions the viability stayed above 80% in comparison to an untreated growth control. The tested groups showed no significant difference between the different time points. Thus, proliferation of the cells is not observably impaired and the impact on the cell viability might be restricted to the moment of laser treatment. A detailed study of the toxicity of GNOME laser transfection is ongoing.

Incubation of ZMTH3 cells with AuNPs with a concentration of 0.5 µg/cm^2^, corresponding to the optimal concentration for GNOME laser transfection, and a ten-fold higher concentration for 3 h did not show a reduction in viability (Fig. 5b). When the incubation time was increased to 24 h a slight decrease in viability could be observed after 48 h, even though it stayed above 90%.

AuNP mediated toxicity is a quite complex field and a large number of conflicting results have been published lately, claiming AuNPs to be toxic [33] or non-toxic [34]. This can be explained by the numerous amounts of parameters impacting the toxicity, as the particle material, size, shape, surface coating and the target cell type. Particles in the size range applied in our study might be taken up into the cells by endocytosis [12], [35], yielding possibly toxic effects. However, Connor et al. demonstrated that internalized particles are non-toxic [34] and Baumgart et al. observed only minor impairment of the cell viability after incubation with comparably high concentrations of 100 nm gold spheres [12]. Therefore, we suppose the AuNPs under the applied conditions as non-toxic. Very small particles in the size range of 1-2 nm might intercalate the DNA and can lead to genetic mutations and long term effects [36]. As such small fragments could arise from the laser-particle interaction, this needs to be evaluated in future studies.

### siRNA transfection and knock down

**Figure 6:**
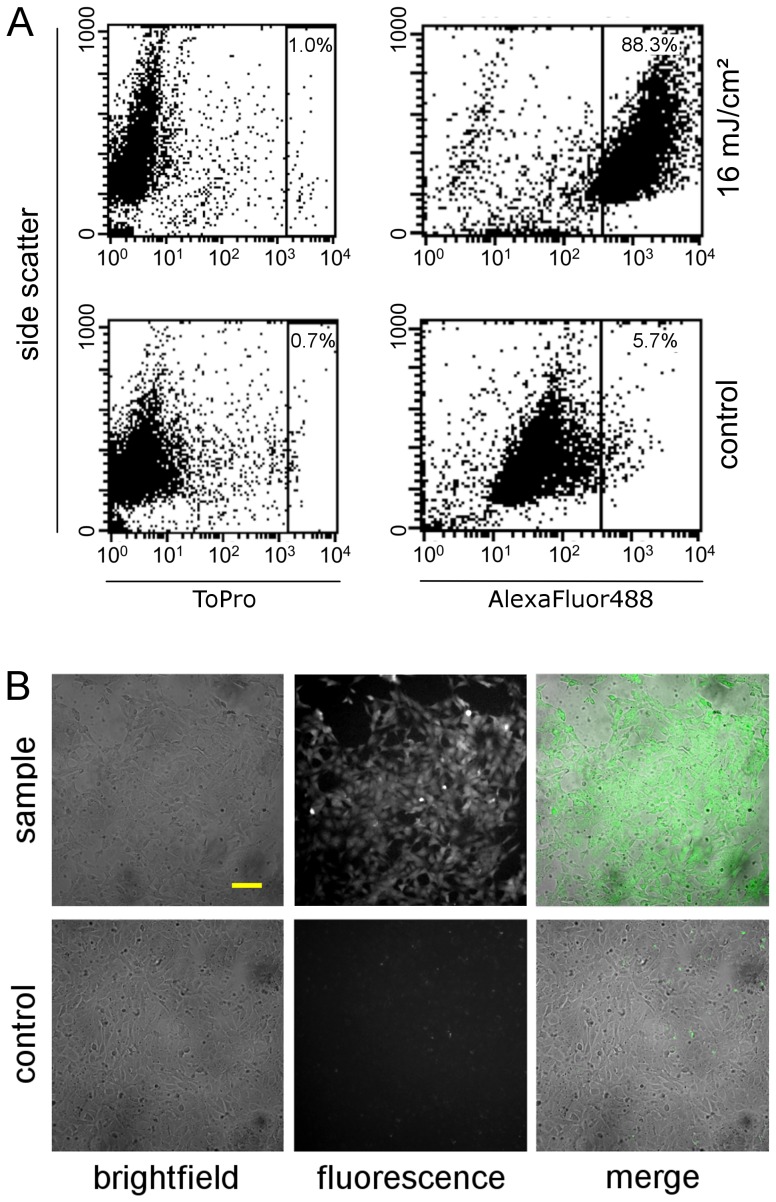
siRNA injection using GNOME laser transfection. A: Transfection of AlexaFluor488 labeled siRNA into ZMTH3 cells. 88% of the cells stained positive for the siRNA after transfection with the optimized parameters (0.5 µg/cm^2^ AuNP, 20 mJ/cm^2^, 50 mm/s). B: Exemplary images of ZMTH3 cells transfected with AlexaFlour488-siRNA and control cells. Scale bar: 100 µm.

Applying the optimized transfection parameters (20 mJ/cm^2^, 50 mm/s, 0,5 µg/cm^2^ 200 nm AuNP), we transfected an AlexaFluor488 labeled siRNA into ZMTH3 cells to analyze the actual transfection efficiency. Using flow cytometry the efficiency was determined to be 88% (Fig. 6a).

To validate the functional knock down, depletion of GFP fluorescence in ZMTH3 cells expressing destabilized EGFP was measured 24 h after laser transfection with anti-GFP siRNA (GFP-22 siRNA, [37]).

**Figure 7:**
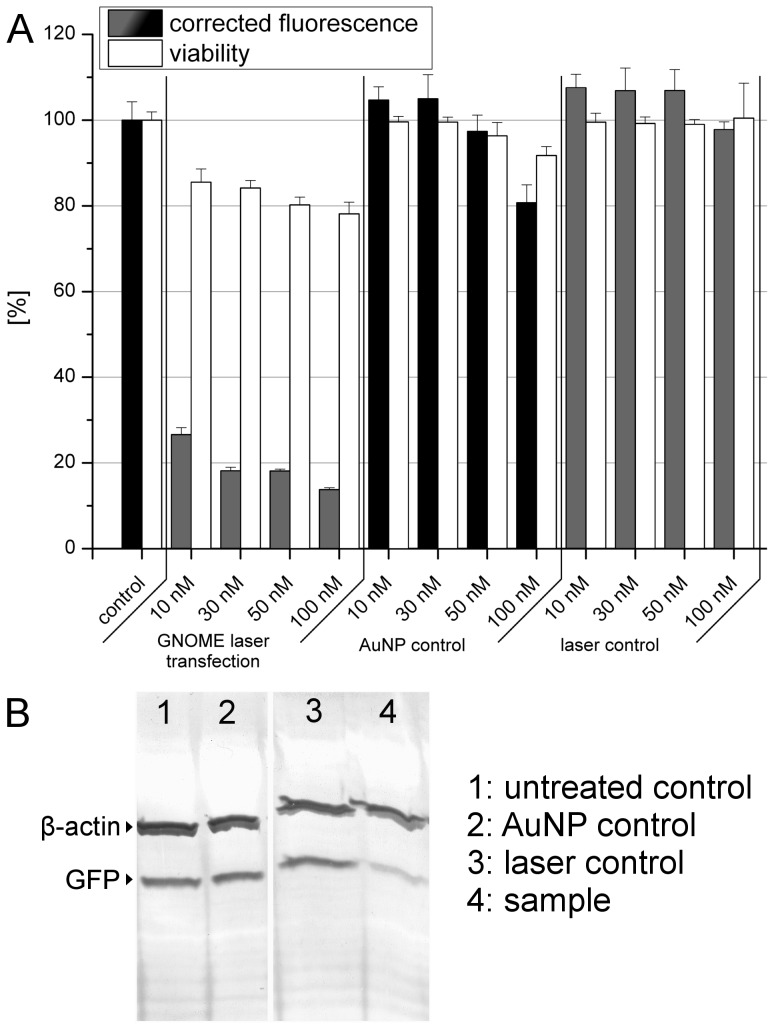
siRNA mediated GFP knock down. A: GFP Fluorescence depletion after GNOME transfection of different siRNA concentrations. No pronounced knock down was observed in the laser and AuNP controls. Values represent the mean of n = 3 experiments +SEM. B: Western blot of β-Actin and GFP after siRNA mediated knock down of GFP. The sample shows a 55% reduction in the GFP band intensity.

The knock down was measured in terms of fluorescence loss compared to an untreated control. To account for cell losses, fluorescence values were additionally corrected according to the corresponding viability as described above. A decrease in fluorescence of up to 82% was achieved (Fig. 7a). Control cells treated with AuNP and siRNA, but not with the laser only showed a minor knock down (19%) for the highest siRNA concentration (100 nM) which is probably linked to the endocytotic uptake of AuNPs. No loss in fluorescence was observed for samples only treated with the laser and siRNA, but without AuNP. To further validate the achieved knock down western blotting was performed (Fig. 7b). A significant reduction of GFP protein level was detected. Semi-quantitative analysis of the GFP band intensity corrected against the corresponding β-actin bands using ImageJ showed a reduction of 55% compared to the untreated control.

### Perforation mechanism

**Figure 8:**
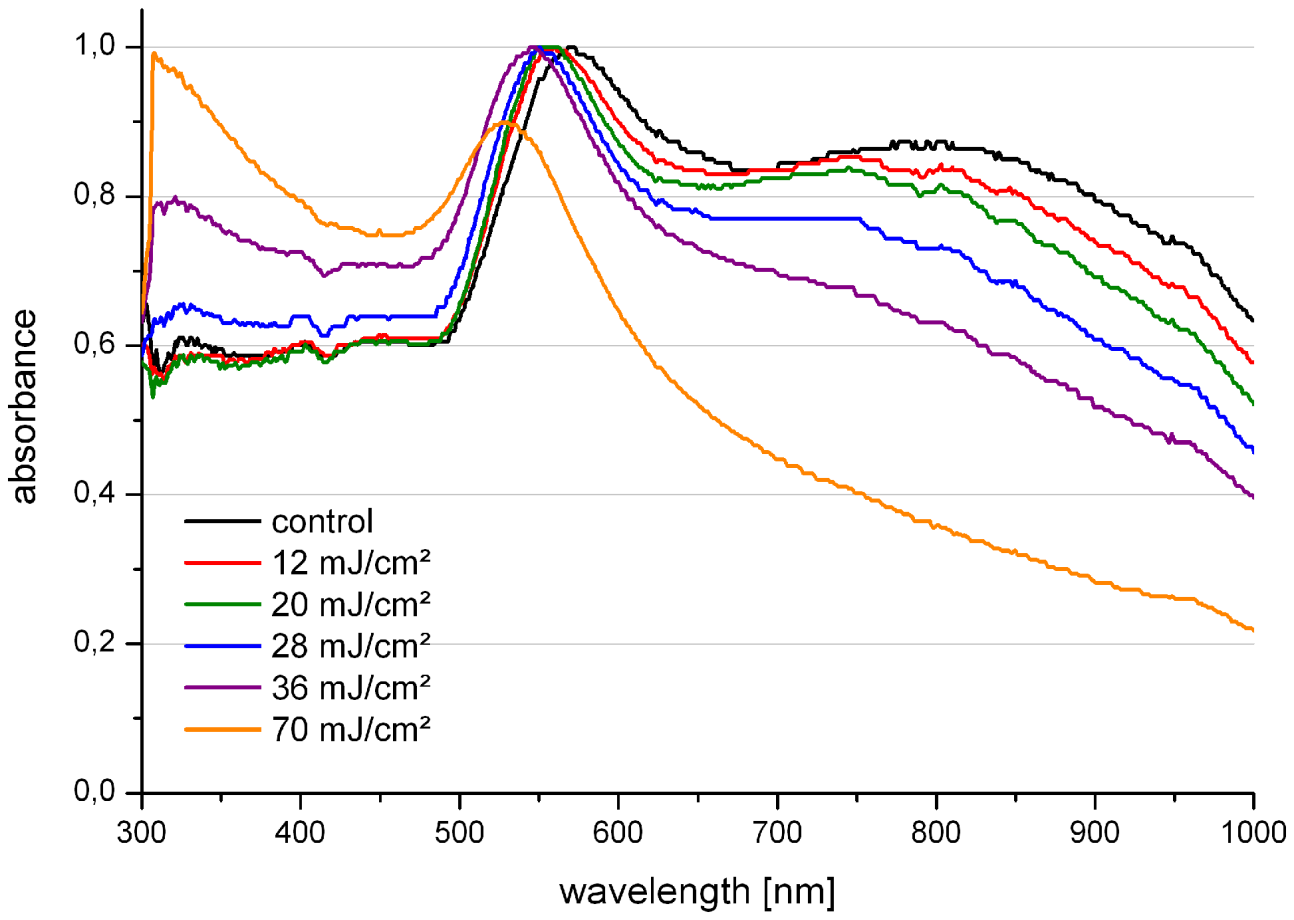
Absorbance spectra of 200 nm AuNP in RPMI after irradiation with different values of radiant exposure.

To determine alterations of the particles after laser irradiation UV/VIS spectra of particles were investigated. Figure 8 shows the spectra of 200 nm AuNPs after irradiation with different radiant exposures. With increasing radiant exposure a decrease of the absorbance shoulder in the near infrared range could be observed. Concurrently, the resonance absorption peak shifted to shorter wavelengths. The exact values of the shifts are presented in Table 1. The UV absorbance increased after radiation with higher radiant exposure. Small alterations in the spectra might be explained by a polishing effect of the laser irradiation: The particle surface starts to melt and as a sphere is the thermodynamic most desirable form, asperities in the surface are removed [29], [38]. With increasing radiant exposure stronger narrowing and blue shifting of the resonance peak occurred as well. This indicates a reduction in the particle diameter, as retardation effects in the particle resonance are less pronounced with decreasing diameters. At higher radiant exposures, fragmentation of the particles can occur. In addition SEM images revealed clusters of melted AuNPs at a radiant exposure of 70 mJ/cm^2^, but no observable change on AuNPs irradiated with 20 mJ/cm^2^ (Fig. S1). These results clearly indicate the existence of thermal effects under the conditions used in this study.

**Table 1:** Shift of the resonance absorbance peak in the absorbance spectra of AuNP after laser irradiation.

Sample [mJ/cm^2^]	Peakshift [nm]	
control	0.0	±0.7
12	−12.6	±0.2
20	−15.4	±0.4
28	−19.3	±1.0
36	−28.3	±1.3
70	−40.6	±1.2

Values represent the mean of n = 3 experiments ±SEM.

We investigated the perforation mechanism in more detail by performing calculations on the particle heating and the near field enhancement with established mathematical models. Temperature calculations utilized the model presented by Liu et al. [39], which is based on the heat transfer model developed by Goldenberg and Tranter [40]. For a 20 mJ/cm^2^ pulse the estimated temperature rise at the particle surface is about 650 K. This is well below the melting point of gold (1337 K). Radiant exposures above 36 mJ/cm^2^ led to a calculated temperature (≥ 1465 K) above the melting point, thus the theoretic values correspond well to our experimental observations. The calculated temperature increase for the transfection conditions would be sufficient to induce protein denaturation, shockwaves due to thermal expansion of the AuNP and water evaporation in close proximity to the particle [28]. All these effects could contribute to membrane perforation, thus supporting a thermal transfection mechanism.

However, a recent study by Kalies and Birr in our lab revealed a non-linear correlation between the threshold pulse energy for a given perforation effect and the number of applied pulses [41]. The corresponding scaling exponent was *kinv ≈* 0.3±0.02 which could be explained well by a three photon ionization of water molecules in close vicinity to the AuNP. On basis of the data presented in Figure 3a, a comparable value of k_inv_ = 0.37 could be calculated (Fig. S2), supporting the theory of a multiphoton mechanism. Based on the Matscat script developed by Schäfer [42]–[44], we calculated the near field enhancement under the conditions used herein to be ∼5.3 (Fig. S3). For the parameters used for GNOME laser transfection (radiant exposure = 20 mJ/cm^2^, spot diameter = 86 µm, pulse length = 850 ps) the intensity of the incident laser light is 2.4×10^7^ W/cm^2^. Hence, an intensity of 1.3×10^8^ W/cm^2^ can be assumed for the near field around the particle. This is well below the predicted threshold for optical breakdown of 6×10^11^ W/cm^2^ for the used parameters [23] and a nanocavitation as reported for gold nanoparticle assisted transfection by femtosecond pulses could be excluded [12], [13].

Our results support both, the appearance of a thermally driven process and possibly multiphoton ionization of (water) molecules as a perforation mechanism. It is likely that at the given parameters both effects occur and support molecular delivery. Wu et al. demonstrated cell membrane perforation upon laser induced AuNP heating [45]. They applied comparable laser parameters, but a seven-fold longer pulsewidth (6 ns) than used in our study, enhancing the contribution of the thermal effects. Since no ablation of the AuNP from the cell surface was observed under GNOME laser transfection conditions (Fig. 4), the appearance of vapour or cavitation bubbles seems to be unlikely as those should lead to particle detachment. Explosive boiling or the generation of plasmonic nanobubbles, as described by Wu et al. [45] and Lukianova-Hleb et al. [16], [46], [47], respectively, therefore is most likely not involved in the perforation mechanism. To gain complete understanding of the mechanism and to distinguish which process is dominant, further investigations are needed.

## Conclusion

The transfection and knock down results presented show that GNOME laser transfection is an efficient technique for the transfection of siRNA and that it can compete with established methods in terms of efficacy and cell viability. Thus, it is a fast and gentle technique for molecular delivery. Our study demonstrates that the effect of single particles in interaction with single laser pulses allows membrane permeabilization. Therefore, high scanning velocities and low AuNP concentrations can be applied while maintaining efficient cell transfection. We found indications for a mixed perforation mechanism consisting of thermal and multiphoton effects in the particle near field. The results provide a strong basis for future investigations and optimization of gold nanoparticle mediated laser transfection.

As other laser based methods already have proven to be applicable to hard to transfect cell types, GNOME is a promising way for antisense applications in primary and stem cells. In future studies it will be of interest, whether these results can be extended to cell types, which are hard to transfect with established methods. Additionally, promising applications of GNOME laser transfection could arise from possible AuNP targeting by antibodies, providing two ways of manipulation selectivity (AuNP binding and spatial selective laser exposure), and the possibility to deliver a large variety of molecules like proteins, Morpholinos and DNA. Within the field of biomedicine, applications in three dimensional cell cultures, tissues or *in vivo* are of special interest and GNOME laser transfection might provide an excellent tool for molecular delivery in these settings. However, such samples could necessitate utilization of near infrared wavelengths to allow deeper laser penetration into the sample and detailed investigations on the AuNP transport into dense cell structures.

## Supporting Information

Figure S1 **Electron microscopical images of 200 nm gold nanoparticles after irradiation with different radiant exposure.** At the highest radiant exposure (70 mJ/cm^2^) melted clusters of particles occur. A: control, B: 20 mJ/cm^2^, C: 70 mJ/cm^2^.
(TIFF)

Figure S2 **The optimal values for radiant exposure for different scanning velocities were plotted against the pulses per point for the given velocity (see also dotted line in** **Fig. 3a** **).** A power function has been fitted to the data. The resulting exponent is b = −0.378. This can be interpreted as a coefficiency of k = 2.65 in the power-law function E_N_ = E_1_*N^(−1/k)^, where E_N_ = threshold pulse energy for N pulses and E_1_ = single pulse threshold energy [48], [49]. Absorption of three photons at a wavelength of 532 nm would yield an energy of 6.99 eV, which is enough to overcome the ionization energy of water (6.5 eV) [50], thus this finding supports the appearance of multiphoton ionization described by Kalies and Birr et al. [41].
(TIFF)

Figure S3 **Calculation of the near field enhancement around a 200 nm gold sphere during irradiation at 532 nm in water.** The color scale represents the electric field enhancement |E|^2^/|E_0_|^2^. The calculation was performed using the MATLAB package developed by Dr. Schaefer (http://www.mathworks.com/matlabcentral/fileexchange/36831-matscat) [43].
(TIFF)
