# Supplementary figures and images for: Gold Nanoparticle Mediated Laser Transfection for Efficient siRNA Mediated Gene Knock Down
Source: PLoS One. 2013 Mar 11;8(3):e58604. doi: 10.1371/journal.pone.0058604 (PMC3594183; doi:10.1371/journal.pone.0058604)

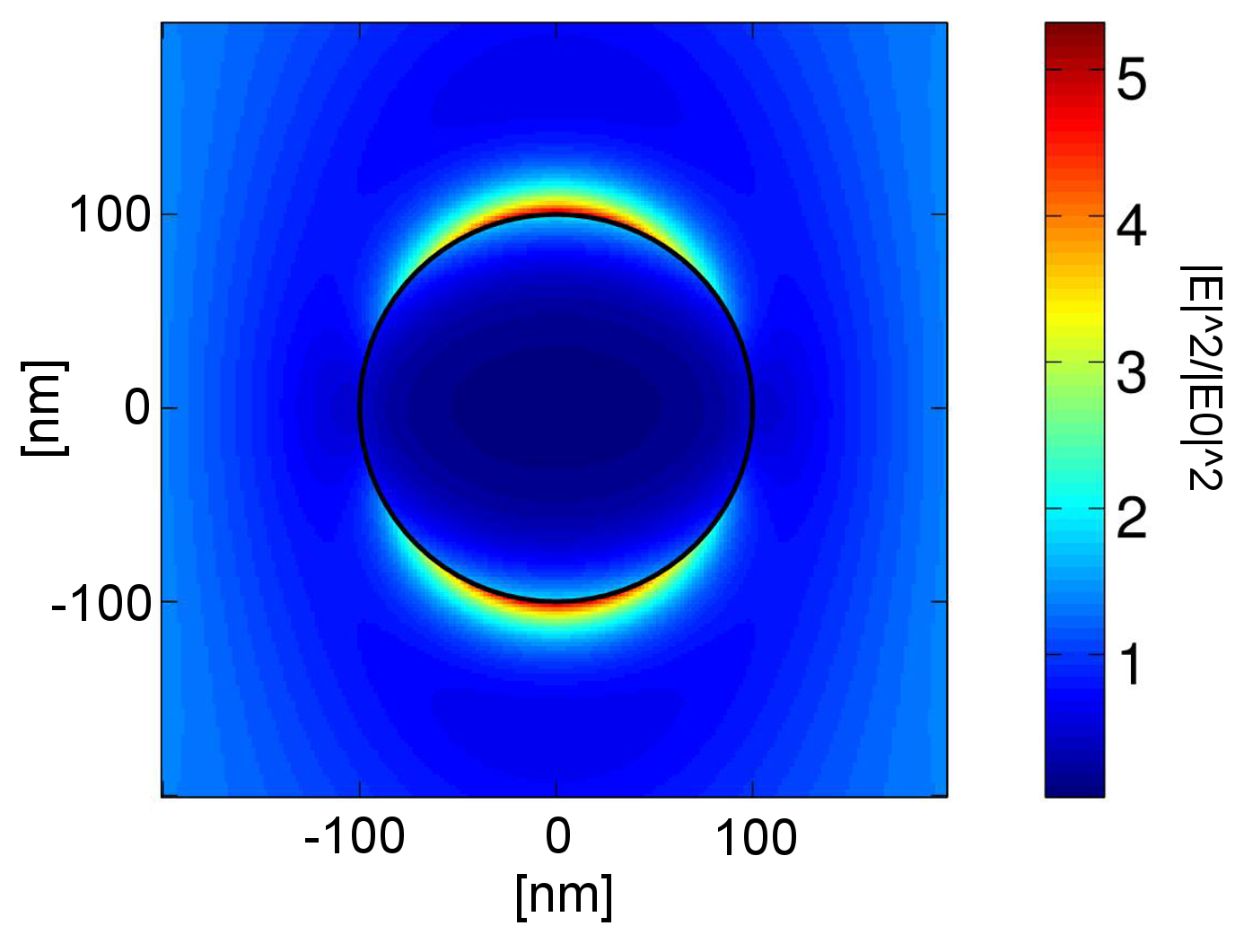

Supplement: Figure S3 — Calculation of the near field enhancement around a 200 nm gold sphere during irradiation at 532 nm in water. The color scale represents the electric field enhancement |E|2/|E0|2. The calculation was performed using the MATLAB package developed by Dr. Schaefer (http://www.mathworks.com/matlabcentral/fileexchange/36831-matscat) [43]. (TIFF) [file pone.0058604.s003.tiff]
